# Autoimmune Thrombotic Thrombocytopenic Purpura: Two Rare Cases Associated with Juvenile Idiopathic Arthritis and Multiple Sclerosis

**DOI:** 10.3389/fmed.2017.00089

**Published:** 2017-07-24

**Authors:** Despoina Dimopoulou, Athina Dimosiari, Eudokia Mandala, Theodoros Dimitroulas, Alaxandros Garyfallos

**Affiliations:** ^1^4th Department of Internal Medicine, Hippokration Hospital, School of Medicine, Aristotle University of Thessaloniki, Thessaloniki, Greece

**Keywords:** multiple sclerosis, juvenile idiopathic arthritis, rituximab, thrombotic thrombocytopenic purpura, autoimmune diseases

## Abstract

Secondary thrombotic microangiopathies are associated with several underlying conditions, with most of them being resolved after the treatment of background disease. Thrombotic thrombocytopenic purpura (TTP) is a rare microangiopathy presenting with anemia, thrombocytopenia, and neurological deficits, occurring most often in various autoimmune diseases due to inhibition of ADAMTS13 by autoantibodies, as well as in pregnant women with or without an autoimmune substrate. In this article, we report two newly diagnosed TTP cases, who have not been published so far. The first is a 27-year-old woman with a history of polyarticular rheumatoid factor negative juvenile idiopathic arthritis, who presented with thrombocytopenia, anemia, schistocytes on blood smear, headache, and active arthritis. Originally she was treated successfully with plasma exchange, intravenous prednisone, and vincristine, and a few months after the TTP episode, she was commenced on rituximab, resulting in remission of primary disease and no relapse of TTP. The second case refers to a 29-year-old pregnant woman complaining of dizziness and fatigue with microangiopathic hemolytic anemia. She was treated with plasma exchanges, intravenous prednisolone, and INN human normal immunoglobulin with full remission of the TTP episode. Six and half years later, she was diagnosed with multiple sclerosis and was commenced on interferon beta-1 alpha, with no recurrent episode of TTP. These cases broaden the spectrum of autoimmune disorders manifested or complicated clinically by TTP. Furthermore, biological agents such as rituximab appear to be an effective treatment option for refractory cases of TTP related to systemic rheumatic disease, indicating an alternative therapeutic solution in persistent cases of this disorder.

## Introduction

Thrombotic thrombocytopenic purpura (TTP) constitutes a rare form of microangiopathic hemolytic anemia, with an incidence of less than 4 cases/million/year. This particular microangiopathy is characterized by the formation of platelet thrombi in the microcirculation (involving capillaries and arterioles), resulting in tissue ischemia and infraction of variable degrees and severity (1). A patient with TTP typically presents with thrombocytopenia, microangiopathic hemolytic anemia of no obvious cause, and schistocytes on blood smears accompanied by a negative direct Coombs test. Clinical symptoms include fever, mild renal failure, and neurological manifestations (2). In view of pathogenic mechanisms, it appears that TTP is associated with decreased activity of von Willebrand factor (VWF) cleaving enzyme, ADAMTS13—a disintegrin and metalloproteinase, with a thrombospondin type 1 motif, member 13—leading to microthrombi formation in the microvascular high sheer environment (3). The decrease in ADAMTS13 can be the result of either congenital deficiency (homozygous or compound heterozygous ADAMTS13 mutations), or more often is attributed to the presence of autoantibodies that inhibit ADAMTS13 or clear it from the blood, accounting for around 95% of TTP cases (4). It is therefore obvious that a TTP episode can occur in the context of various autoimmune diseases, a good number of which have been reported in association with TTP (5, 6). While predisposition relationship between pregnancy and TTP has been demonstrated, it remains unknown whether the higher incidence of TTP in pregnant women is associated with the pregnancy itself or predisposes to the development of a systemic autoimmune disease (7). From the therapeutic point of view, plasma exchange is the gold standard for the treatment of TTP achieving survival rates up to 80% (8). Recently, the initiation of biologic regimens in the management of autoimmune disease has partially changed the approach to TTP especially in patients with underlying autoimmune dysregulation indicating rituximab—a chimeric monoclonal antibody targeted against the pan-B-cell marker CD20 as a reliable, safe treatment option in combination with plasma exchange and corticosteroids.

In this report, we present two cases of TTP, the first in a patient with polyarticular rheumatoid factor (RF) negative subtype of juvenile idiopathic arthritis (JIA) and the second in a patient who developed multiple sclerosis (MS) a few years following the TTP episode. Both individuals gave their written informed consent according to the Declaration of Helsinki patients.

## Case Reports

### Case 1

A 27-year-old woman with a 21-year history of polyarticular RF negative JIA was admitted to the emergency department, complaining of general malaise and strong headache. On the top of these symptoms, she also reported symmetric polyarthritis involving small joints of the hands, wrists, elbows, and ankles. At the time of admission, the patient was on methotrexate 12.5 mg per week, prednisone 5 mg per day and adalimumab 40 mg every 15 days. Initial physical examination revealed a body temperature of 37.1°C, heart rate 115 bpm, blood pressure 116/80 mmHg, and active symmetric polyarthritis arthritis. Specifically, her joints were swollen, acutely tender to palpation and painful in movement, erythrocyte sedimentation rate was elevated at 94 mm/h, clinical and biochemical findings translated into disease activity score 28 of 6.1. At that point, no rash, lymphadenopathy, or hepatosplenomegaly were noticed. Initial laboratory testing revealed Ht 23.7%, Hb 8.4 g/dl, white blood cell count of 15,690/mm^3^, platelet count 30,000/mm^3^, LDH 1,070 U/L, total bilirubin 3.2 mg/dl, indirect bilirubin 2.9 mg/dl, AST 35 U/L, ALT 27 U/L, albumin 4.4 g/dl, BUN 43 mg/dl, creatinine 0.79 mg/dl, CRP 95 mg/dl (<5 normal values) and fibrinogen, prothrombin and activated partial thromboplastin times (PT, aPTT) were normal. Direct Coomb’s test was negative but reticulocytes 10% and schistocytes 10–12 per high-power field were observed on peripheral blood smear. Urinalysis was normal. All serological tests for hepatitis B, hepatitis C, HIV, Lyme disease, brucellosis, rickettsial infection, Parvo B-19, cytomegalovirus were negative, as well as tumor markers. Blood, urine, and stool cultures were also negative and disseminated intravascular coagulation was excluded. Pregnancy test using urine sample was negative. Immunological profile including RF, anti-nuclear antibodies, anti-double-stranded deoxyribonucleic acid antibodies, extractable nuclear antigens screen, antineutrophil antibodies, anti-cardiolipin, antib2GPI, as well as anti-cyclin citrullinated peptide was negative. C3 level was elevated and C4 value was normal. All specimens were collected on admission and prior to initiation of therapeutic intervention. A thoracic radiography and computed tomography of the brain was performed with no specific findings. On the basis of microangiopathic hemolytic anemia, thrombocytopenia, and headache, the patient was diagnosed with TTP on the background of active JIA and adalimumab was withdrawn. She was treated with plasma exchange and intravenous prednisone 100 mg daily. Vincristine 2 mg/week for 2 weeks was added. After 10 daily plasma exchanges, her platelet count increased gradually from 30,000 to 285,000/mm^3^, while her LDH level decreased from 1,070 to 339 U/L. The patient’s symptoms improved gradually and she was discharged in good condition. The following months her hemoglobin remained low with an elevated LDH, simultaneously suffering from many episodes of active polyarthritis. Nine months after her hospitalization, it was decided to discontinue methotrexate and initiate treatment with rituximab, receiving two cycles of 1,000 mg, while progressively tapering oral corticosteroids. In a yearly follow-up, there has been no relapse of TTP, while patient’s arthritis remains in remission.

### Case 2

A 29-year-old woman, 31 weeks pregnant, was admitted to the emergency department with intense dizziness and fatigue. She has a history of retrobulbar neuritis on the postpartum period of a previous pregnancy, with no further complications and investigations. Physical examination revealed a normal blood pressure of 110/75 mmHg, body temperature 36.5°C. Emergency fast ultrasound proved a dead fetus. Laboratory work up pointed toward the direction of microangiopathic hemolytic anemia (Ht 19%, Hb 6.9 g/dl) with 12–14 schistocytes per high-power field observed on peripheral blood smear, alongside with thrombocytopenia (platelet count 13,000/mm^3^), LDH 832 U/L, negative direct Coombs reaction and normal rates of liver enzymes, renal biochemistry, and PT, PTT. Blood and urine cultures were also negative, as well as all serological work ups, including HBV, HCV, HIV, EBV, CMV, Rubella, Toxoplasma, and Herpes Simplex. Finally, the patients’ immunological profile was also inclusive negative. An urgent C-section was performed, with the patient presenting postoperatively worsening of her hematological profile, with the clinical addition of persistent intense headache. Given the type of anemia, the thrombocytopenia, and central nervous system symptomatology, the suspicion of TTP was raised and the patient was treated with plasma exchanges, intravenous prednisolone 100 mg daily, and intravenous INN human normal immunoglobulin 30 g for 5 days. She fully recovered but 3 months later she presented with a new episode of TTP, successfully responding to the same treatment. Six and half years later, a period of time in which our patient remained free of TTP relapses and with no other pregnancy or miscarriage occurring, she presented with progressive myelopathy. Magnetic resonance imaging of brain and neck showed signs consistent with active MS, suggesting that the past—probably underappreciated—episode of retrobulbar neuritis was most likely to represent initial, unrecognized presentation of MS—brain magnetic resonance imaging and other diagnostic assessment had not been performed at that stage. She was started on interferon beta-1 alpha cytokine, and 14 years later, she has not suffered with any episode of TTP.

## Discussion

In the current paper, we present two TTP cases in individuals with systemic disease. Although the pathogenetic model of TTP in both congenital and acquired cases is the diminished VWF cleavage, difficulties in the measurement of ADAMTS13 antigen and antibody, usually lead to a clinical diagnosis based on laboratory findings consistent with microangiopathitic anemia. Due to the lack of technical support, both of our cases were diagnosed according to clinical perspective and characteristic laboratory tests (Table 1).

**Table 1:** Main laboratory and clinical findings of TTP cases at the time of diagnosis.

	Case 1	Case 2
History of autoimmune disease before TTP episode	RF negative JIA	None
CNS symptoms	Headache	Dizziness
Body temperature (°C)	37.1	36.5
Hb (14–18 g/dl)	8.4	6.9
Platelet count (150,000–400,000/mm^3^)	30,000	13,000
Peripheral blood smear	Schistocytes	Schistocytes
Coagulation^a^	Normal	Normal
Direct Coomb’s	Negative	Negative
LDH (150–450 U/L)	1,070	832
Virology^b^	Negative	Negative
Immunological profile^c^	Negative	Negative
Liver bichemistry	Normal	Normal
Blood, urine, stool cultures	Negative	Negative

*TTP, thrombotic thrombocytopenic purpura; CNS, central nervous system; Hb, hemoglobin; LDH, lactic dehydrogenase; RF, rheumatoid factor; JIA, juvenile idiopathic arthritis*.

*^a^PT, aPTT, INR, fibrinogen*.

*^b^Hepatitis B and C virus, Epstein–Barr virus, Herpes Simplex virus, cytomegalovirus, human immunodeficiency virus*.

*^c^RF, anti-nuclear antibodies, anti-double-stranded deoxyribonucleic acid antibodies, extractable nuclear antigens screen, antineutrophil antibodies, anti-cardiolipin and antib2GPI, anti-cyclin citrullinated peptide*.

Several autoimmune diseases have been reported in association with TTP (Table 2), but to the best of our knowledge, these are extremely rare cases described in JIA or MS patients. Particularly in view of JIA, there is no reported case in literature. Both patients described in this paper had negative autoimmune screen—performed prior to the initiation of plasma exchange—which from one side may underline the rarity of our cases and from the other indicate that co-existence of two autoimmune conditions is also possible. In this respect, one point to be addressed is that the treatment of TTP itself might mask immunological changes by decreasing serum levels of autoantibodies. Subsequently, it is important to assess such abnormalities before any therapeutic intervention, should the patients’ condition allow extensive diagnostic workout (9). On the other hand, a variety of autoimmune disorders may develop several years after the recovery of TTP as indicated by the MS case and such observations highlight the necessity of clinical surveillance. Although guidelines are missing, a high index of suspicion in these persons accompanied by a periodic follow-up—ideally by a combined team of hematologists, rheumatologists, and general practitioners—could contribute to the prompt identification of symptoms and the initiation of proper treatment early in the course of the disease. The acknowledgment of this possible complication across different medical specialties is also important for early diagnosis.

**Table 2:** Systemic autoimmune diseases associated with thrombotic thrombocytopenic purpura.

Systemic lupus erythematosus (9)
Rheumatoid arthritis (9)
Dermatomyositis (9)
Scleroderma (9)
Antiphospholipid syndrome (10)
Mixed connective tissue disease (11)
Primary Sjogren syndrome (12)
Psoriasis (12)
Ankylosing spondylitis (12)
Sarcoidosis (12)
Primary biliary cirrhosis (12)
Multiple sclerosis (1)
Inflammatory bowel disease (12)

Low baseline ADAMTS13 activity of 10%, regardless to detection of anti-ADAMTS13 autoantibodies, strongly supports the diagnosis of TTP in a patient with thrombocytopenia and microangiopathitic anemia providing that a detailed evaluation has ruled out other causes of thrombotic microangiopathy (13, 14). Given the relatively low sensitivity in the assessment of ADAMTS13 levels, treatment options and decisions should be based on clinical features and laboratory findings, as well as patient’s history and not on ADAMTS13 title results as occurred in our reported cases (15–17).

As in non-pregnant patients, the vast majority of TTP cases in pregnancy are acquired, with a low ADAMTS13 antigen activity and IgG anti-ADAMTS13 measured in serum. In congenital cases, there is no detectable antibody, while most women appear to be *nulliparous* (18, 19). Although there are no significant differences in the clinical presentation, TTP outcome of the pregnancy depends on etiology. Hereditary ADAMTS13 deficiency is associated with fetal loss, preeclampsia, HELLP syndrome, TTP, or atypical hemolytic uremic syndrome. Moreover, the outcome is strongly related to the trimester on occurrence, with the second one presenting with worst outcome (fetal loss) (19, 20). Differentiating congenital from acquired episodes is also important in means of therapeutic intervention, with plasma exchanges being the basic treatment in the former and immunosuppressive therapy being essential in the later (21, 22).

Thrombotic thrombocytopenic purpura is a very serious rapidly deteriorative disease, leading to early death if not drastically treated within hours from admission. The most critical therapeutic intervention remains plasma exchange, especially when initiated immediately, directly removing autoantibodies and supplementing the activity of ADAMTS13 (22, 23). In view of concomitant interventions aiming to limit plasma exchange sessions required to achieve remission, various approaches have been used, predominantly corticosteroids as monotherapy or combined with other immunosuppressants such as cyclophosphamide, vincristine, cyclosporine and azathioprine; in uncommon refractory cases, splenectomy still represents an alternative therapeutic option (23). Over the last decade, novel therapeutic approaches such as monoclonal antibodies and biologic agents have achieved remarkable therapeutic outcomes by inhibiting immune cell activation. Rituximab is widely used in lymphoproliferative disorders and many autoimmune diseases such as rheumatoid arthritis, systemic lupus erythematosus, and scleroderma (24). Interestingly, it appears to be extremely efficacious in hematologic complications of autoimmune diseases including TTP, idiopathic thrombocytopenic purpura, and even catastrophic antiphospholipid syndrome (25, 26). With regard to TTP, rituximab destroys anti-ADAMTS13-producing B cells and suppresses excessive cytokine production, consisting through these pathways to a promising first line treatment of TTP (27, 28). Additionally, evidence show that preemptive injections of rituximab in severe cases of TTP reduced the incidence of relapse, continually restoring the activity of ADAMTS13 and suppressing anti-ADAMTS13 antibodies, suggesting a role for rituximab as maintenance treatment, with reasonable toxicity (29–31).

## Concluding Remarks

We presented two cases of TTP, one in polyarticular RF negative subtype of JIA and the other in MS, diseases that should be taken into account in individuals presented with this severe form of microangiopathy. The awareness of such combination is important in the daily clinical practice although the likelihood of these conditions occurring in the same patient is quite rare and can commonly reflect distinct autoimmune processes irrelevant to each other. However, therapeutic interventions aiming to B-cell depletion and reduction of autoantibodies, with rituximab, appear very effective both as induction therapy for the initiation of remission, as well as maintenance therapy.

## Author Contributions

AD, DD, and TD prepared the body of the manuscript. DD and EM made the diagnosis, performed the diagnostic and laboratory workout, and treated the patients. AG critically reviewed the publication. All the authors endorsed the final form of the manuscript.

## Conflict of Interest Statement

The authors declare that the research was conducted in the absence of any commercial or financial relationships that could be construed as a potential conflict of interest. The reviewer NV and the handling Editor declared their shared affiliation, and the handling Editor states that the process nevertheless met the standards of a fair and objective review.
